# Successful Treatment of Chronic Lymphocytic Leukemia Multifocal Central Nervous System Involvement with Ibrutinib

**DOI:** 10.4274/tjh.2017.0313

**Published:** 2018-05-25

**Authors:** Anna Christoforidou, Georgios Kapsas, Zoe Bezirgiannidou, Spyros Papamichos, Ιoannis Kotsianidis

**Affiliations:** 1 Democritus University of Thrace, Department of Hematology, Alexandroupolis, Greece; 2 Democritus University of Thrace, Department of Radiology, Alexandroupolis, Greece

**Keywords:** Chronic lymphocytic leukemia, Central nervous system, CNS, Ibrutinib

**To the Editor,**

Central nervous system involvement (CNSi) is rare in the course of chronic lymphocytic leukemia (CLL). The frequency ranges from 0.8% to 1% [1], and it is often underreported. Diagnosis is challenging and there is no consensus on the optimal therapy or survival. CNSi manifests as either leptomeningeal infiltration or a focal parenchymal lesion, or both [1]. We describe the case of a CLL patient who progressed with parenchymal CNS involvement and was successfully treated with ibrutinib.

A 71-year-old woman was followed without treatment at the hematology clinic for 12 years for asymptomatic CLL, Binet stage I, exhibiting slowly progressive lymphocytosis and mild hepatosplenomegaly. In March 2016 she presented with expressive aphasia, memory problems, confusion, and headache, but no B symptoms. Neurological examination confirmed the mental and speech impairment but was otherwise unremarkable. Thoracic and abdominal computed tomography scan showed no lymphadenopathy or progression of visceromegaly. Her complete blood count was unchanged compared to the previous year with WBC lymphocytes at 14,652x10^9^/L, Htc at 45%, and platelets at 144x10^9^/L, with typical CLL morphology and immunophenotype (CD19 83% with CD5+/CD23+/CD20+low/CD38-/sIglow) and unmutated p53. IGH mutational analysis showed a mutated clone with IGHV3-7/IGHD1-26/IGHJ4 rearrangement. Serum chemistry was normal apart from elevated lactate dehydrogenase at 303 U/L (upper normal limit: 248 U/L). Antinuclear antibody and rheumatoid factor were negative; C-reactive protein, C3, and C4 levels were within the normal limits. Magnetic resonance imaging (MRI) showed a contrast-enhanced irregularly shaped mass of 22x17x16 mm in the left frontal lobe with intense edema and midline shift (Figure 1A). Lumbar puncture showed 5/µL nucleated cell count, 5/µL erythrocytes, 0.4 g/L protein, and no monoclonal B lymphocytes (CD5/CD19) by flow cytometry. Extensive investigations for infection with cytomegalovirus, Epstein-Barr virus, human immunodeficiency virus, herpes simplex virus, ortoxoplasma antibodies as well as PCR for *Cytomegalovirus* DNA were negative in both serum and cerebrospinal fluid. She was referred to a neurosurgeon but the patient was reluctant to undergo a core biopsy of the brain lesion. However, dynamic susceptibility contrast MRI perfusion imaging displayed a signal intensity curve overshooting above the baseline that was suggestive of lymphoma (Figure 1G) [2].

Considering the above findings, the patient was treated in an exploratory fashion with rituximab plus a high-dose methylprednisolone (RHDM) regimen (rituximab at 500 mg/m^2^ i.v. and methylprednisolone at 1g iv for 4 days). After 2 monthly cycles, the neurological symptoms partially regressed, but her MRI findings deteriorated with a new lesion on the left frontal lobe, although the original lesion was impressively smaller (Figures 1B and 1C). Continued RHDM resulted in a decrease of lymphocytosis to 10.9x10^9^/L, but repeat MRI showed an atypical pattern of older lesions receding coupled with the appearance of new ones in multiple cerebral sites (Figure 1D). Since we did not have proof of whether the infiltrating neoplastic cells were identical to the original leukemic clone or a manifestation of Richter’s syndrome (RS), second-line treatment was a challenge. The patient was switched to ibrutinibat 420 mg per day, based on the recent reports of ibrutinib’s CNS penetration and effectiveness, even in high-grade lymphomas. Three months later there was a partial improvement in the MRI findings and no new lesions. Currently on the 15^th^ month of ibrutinib therapy, she is completely symptom-free , shows partial response of CLL and stable neuroimaging improvement, 21 months after initial CNS involvement (Figure 1E, 1F). 

Autopsy studies have found leukemic meningitis and parenchymal brain involvement in up to 20% of CLL patients, but clinical syndromes are very rarely reported [3], with the first ever case published by Solal-Celigny et al. [4]. CNSi is diagnosed by neuroimaging, cerebrospinal fluid evaluation, and core tissue biopsy that differentiate between CLL, Richter’s transformation, or another solid tumor. Strati et al. [1] reviewed 33 patients with CLL CNSi and, among them, 11 out of 12 patients with CNS RS had later developed systematic disease [1]. Our patient did not at any point develop systematic Richter’s syndrome and has an excellent clinical course during the 21 months of follow up which is suggestive of a CLL rather than RS origin of the CNSi.

The treatment outcome of clinically apparent CNSi is unclear, as most studies are retrospective. The management ranges from CLL therapy alone [5] to CNS irradiation, intrathecal chemotherapy, and intensive CNS-lymphoma modalities. Intrathecal rituximab has been used in several case reports and in a small study for high-grade CNS lymphomas but never in CLL [6]. In a recent study the median overall survivalof patients with CLL or RS brain involvement was 12 and 11 months, respectively [1]. On the contrary, a cohort of 30 French patients had much better overall survivalof 65% at 5 years [7]. Ibrutinib is an oral Bruton tyrosine kinase inhibitor approved for B-CLL [8]. It is a small molecule that crosses the blood-brain barrier with promising results in CNS lymphoma, as shown in some cases of mantle cell lymphoma [9,10,11], in Waldenström macroglobulinemia patients [12,13,14], and, more importantly, in a phase I study of 20 patients with relapsed/refractory CNS lymphoma showing 75% overall response rate, including 8 complete responses, although responses were relatively short-lived [15]. Ibrutinib has a convenient outpatient oral administration scheme with minimal toxicity and is an attractive option for CNS lymphoma compared to traditional intensive chemotherapy and/or intrathecal therapy.

So far, there are seven published cases of CLL with CNSi treated with ibrutinib monotherapy (Table 1): two with nodular masses [7,16], four with leptomeningeal disease [7,16], and one with cervical myelopathy [17]. None of these patients underwent brain biopsy. All patients received the standard dose of 420 mg/day and all of them responded with sustained complete responseor partial response, with a median follow-up of 8 to 18 months. Our patient had multiple brain masses and shows an ongoing response to second line ibrutinib monotherapy for a total of 21 months as per December 2017, when the latest brain MRI was performed.

In conclusion, this case further supports the efficacy of ibrutinib in CLL with CNSi, suggesting a potential future change in the frontline management and also the outcome of this rare condition. 

## Figures and Tables

**Table 1:**
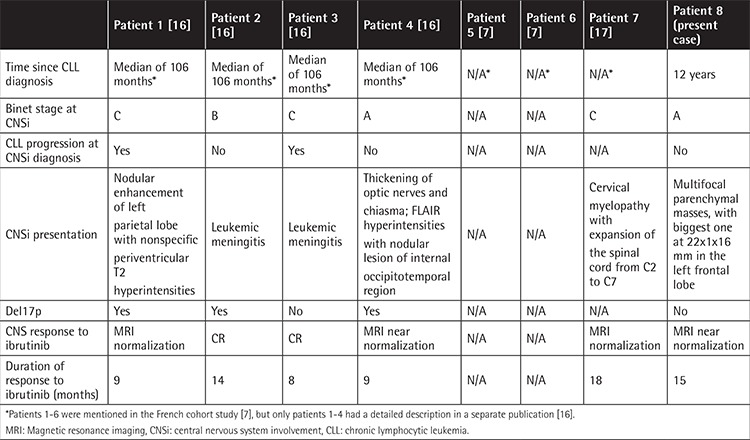
Characteristics of published cases of ibrutinib-treated chronic lymphocytic leukemia central nervous system involvement

**Figure 1:**
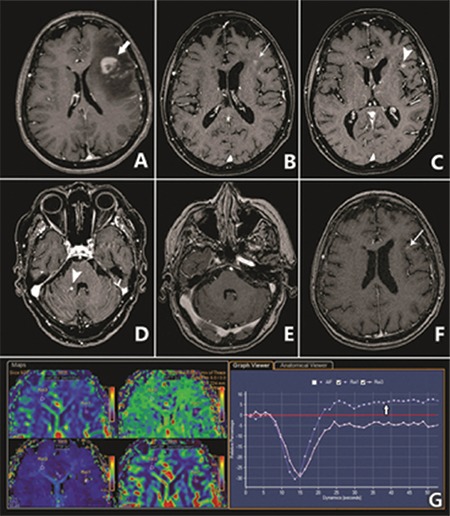
A) Initial presentation of the enhancing lesion in the left frontal lobe (thick arrow), with considerable perilesional edema. B, C, D) After one and four rituximab plus a high-dose methylprednisolone cycles there was a reduction of the enhancing lesion (thin arrow) and edema; however, new enhancing lesions appeared in the left frontal operculum and the right middle cerebellar peduncle (arrowheads). E) Brain magnetic resonance imaging 5 months after ibrutinib therapy demonstrates complete resolution of the cerebellar lesion and F) minimal enhancement in the area of the lesion in the left frontal operculum (arrow). G) Dynamic susceptibility contrast perfusion imaging. Comparison between the enhancing lesion and the normal contralateral side demonstrates an overshooting of the intensity curve of the lesion above the baseline (arrow). This phenomenon is suggestive of lymphoma [149x172 mm (72x72 DPI)].
